# Exposure of Microglia to Interleukin-4 Represses NF-κB-Dependent Transcription of Toll-Like Receptor-Induced Cytokines

**DOI:** 10.3389/fimmu.2021.771453

**Published:** 2021-11-22

**Authors:** Ella A. Zuiderwijk-Sick, Céline van der Putten, Raissa Timmerman, Jennifer Veth, Erica M. Pasini, Linda van Straalen, Paul van der Valk, Sandra Amor, Jeffrey J. Bajramovic

**Affiliations:** ^1^ Alternatives Unit, Biomedical Primate Research Centre, Rijswijk, Netherlands; ^2^ Department of Parasitology, Biomedical Primate Research Centre, Rijswijk, Netherlands; ^3^ Department of Pathology, Vrije Universiteit (VU) Medical Centre, Amsterdam, Netherlands

**Keywords:** macrophages, microglia, glioma, innate immunity, toll-like receptor, alternative activation

## Abstract

Interleukin (IL)-4 is a cytokine that affects both adaptive and innate immune responses. In the central nervous system, microglia express IL-4 receptors and it has been described that IL-4-exposed microglia acquire anti-inflammatory properties. We here demonstrate that IL-4 exposure induces changes in the cell surface protein expression profile of primary rhesus macaque microglia and enhances their potential to induce proliferation of T cells with a regulatory signature. Moreover, we show that Toll like receptor (TLR)-induced cytokine production is broadly impaired in IL-4-exposed microglia at the transcriptional level. IL-4 type 2 receptor-mediated signaling is shown to be crucial for the inhibition of microglial innate immune responses. TLR-induced nuclear translocalization of NF-κB appeared intact, and we found no evidence for epigenetic modulation of target genes. By contrast, nuclear extracts from IL-4-exposed microglia contained significantly less NF-κB capable of binding to its DNA consensus site. Further identification of the molecular mechanisms that underlie the inhibition of TLR-induced responses in IL-4-exposed microglia may aid the design of strategies that aim to modulate innate immune responses in the brain, for example in gliomas.

## Introduction

Interleukin (IL)-4 is well known for its biological functions during humoral and cell-mediated adaptive immune responses. It stimulates the proliferation of activated B and T cells, it is pivotal for the differentiation of B cells into plasma cells and it skews T cell-mediated cytokine production (1). In addition, IL-4 exerts effects on cells of the innate immune system. It is important for the differentiation of monocyte-derived dendritic cells (DC), it enhances MHC class II expression levels and it promotes the alternative activation of macrophages, while inhibiting classical activation (2, 3).

In the central nervous system (CNS), IL-4 is present during homeostatic and pathological conditions (4–6). Its production can be induced in astrocytes and microglia (7–9), that both have been reported to express IL-4 receptors (IL-4R) as well (5, 6, 10, 11). IL-4 plays a role in neuroinflammatory diseases like viral encephalitis and multiple sclerosis, but has also been implicated in the development and progression of gliomas. Different gliomas secrete IL-4, and glioblastoma multiforme (GBM) risk and outcome are correlated to polymorphisms in IL-4R gene loci (12–15). In addition, it has been described for many tumor types that IL-4 can induce macrophages to support tumor survival by creating an immunosuppressive environment (16–18).

Microglia are the resident macrophages of the brain and are major players in innate and adaptive immune responses during CNS injury or disease (19–21). Although microglia share ontological precursors with myeloid cells such as monocytes, macrophages and DC, they originate from a distinct progenitor (22). Different studies have addressed the plasticity of microglia after exposure to IL-4. Whereas neonatal rodent microglia have the capacity to differentiate into DC (23), more recent studies using fetal and adult human microglia demonstrate that these cells are less plastic (24). In line with this, exposure of adult human microglia to IL-4 rather induces the expression of cellular markers that are associated with alternative activation of macrophages and endows them with anti-inflammatory properties (24). However, when compared to blood-derived macrophages, microglia acquire distinctively different properties. These include differences in their expression levels of cell surface molecules, in their capacity to phagocytose myelin as well as in their innate immune responses such as the profile of TLR4-induced cytokine responses (25).

Earlier work from our lab demonstrated that exposure of adult rhesus microglia to other differentiation inducing stimuli affects innate immune responses (26). Here, we studied the effects of IL-4 on microglial innate immune responses in detail, profiled microglial cell surface protein expression levels and assessed their potential to present antigen. We demonstrate that TLR-induced cytokine responses were broadly impaired at the transcriptional level in IL-4-exposed microglia, and that IL-4 type 2 receptor-mediated signaling was critically involved. In addition, we show that nuclear translocalization of nuclear factor (NF)-κB upon LPS exposure was intact in IL-4-exposed microglia, and that differential expression of epigenetic modulators was also unlikely to explain the impaired LPS-induced cytokine responses in IL-4-exposed microglia. Finally, we demonstrate that in nuclei of IL-4-exposed microglia, LPS-induced binding of NF-κB to target DNA is reduced. These findings may help to understand how innate immune responses are inhibited mechanistically in microglia, and may aid therapeutic approaches aimed at recruitment of innate immune responses.

## Materials and Methods

### Animals

Adult brain donor rhesus monkeys (*Macaca mulatta*) without neurological disease became available from the out bred breeding colony at the Biomedical Primate Research Centre (BPRC); no monkeys were sacrificed for the exclusive purpose of primary cell culture initiation. Individual details are listed in **Supplementary Table 1**. Better use of experimental animals contributes to the active 3Rs program within the BPRC.

### Primary Cell Isolation and Culture

Primary microglia were obtained from adult rhesus monkeys at necropsy as described previously (26). Cells were plated at a density of 2.2-2.5x10^5^/ml in tissue culture-treated 6 or 24-well plates (Corning Costar Europe, Badhoevedorp, The Netherlands) in microglia medium (1:1 v/v DMEM (high glucose; Life Technologies, Breda The Netherlands)/HAMF10 (with L-glutamine; Life Technologies) with 10% (v/v) FCS, 2 mM glutamax and antibiotic supplement [penicillin 100 U/ml and streptomycin 0.1 mg/ml (all Gibco, Life Technologies)] supplemented with ≥ 4 units recombinant human MCSF/ml, ≥ 40 units recombinant human GMCSF/ml or ≥ 40 units recombinant human GMCSF/ml + 200 ng recombinant human IL-4/mL or IL13/mL (all Peprotech, London, UK). Half of the medium was replaced by fresh medium containing new growth factors every 3-4 days.

Peripheral blood mononuclear cells (PBMCs) were isolated from rhesus monkey donors at necropsy using lymphocyte separation medium (LSM, MP biomedicals, Santa Ana, CA) gradient centrifugation. Mononuclear cells were isolated with anti-CD14 monoclonal antibody-coated Microbeads using MACS single-use separation columns from Miltenyi Biotec (Bergisch Gladbach, Germany) as described by the manufacturer. Purified CD14+ cells were resuspended in RPMI (Gibco; Life Technologies) containing 10% (v/v) FCS, 2 mM glutamax and penicillin 100 U/ml and streptomycin 0.1 mg/mL (all Gibco), supplemented with ≥ 4 units recombinant human MCSF/ml or ≥ 40 units recombinant human GMCSF/ml + 200 ng recombinant human IL-4/mL (all Peprotech) to yield macrophages or DCs respectively. Half of the medium was replaced by fresh medium containing new growth factors every 3-4 days.

### Antibodies and Reagents

For flow cytometrical analyses the following antibodies were used: CD11b-APC (clone D12, mouse IgG2a, BD, Alphen a/d Rijn, the Netherlands), CD11c-APC (clone S-HCL-3, mouse IgG2b, BD), CD14-APC (clone M5E2, mouse IgG2a, BD), CD45-PerCP (clone Tu116, mouse IgG1, BD), CD83 (clone HB15a, mouse IgG2b, Beckman Coulter, Woerden, the Netherlands), CD86-FITC (clone BT-7 mouse IgG, Diaclone, Besancon, France), HLAdr–PerCP and APC (clone L243, mouse IgG2a, BD), HLA-ABC (clone G46-2.6, mouse IgG1, BD), TLR2 (TL2.1, mouse IgG2a, Biolegend, San Diego, CA), TLR4 (HTA125, mouse IgG2a, Biolegend), TREM2 (clone 263602, mouse IgG2b, Jackson lab, Suffolk, UK), Foxp3-APC (PCH101, Rat IgG2a, eBioscience, San Diego, CA), goat anti mouse-FITC (BD). Isotypes controls were all from BD, except the rat IgG2a-APC isotype control (eBioscience). For immunofluorescence microscopy the following antibodies were used: anti-TREM2 (mouse IgG2b, Jackson lab), anti-NF-κB (rabbit IgG, Santa Cruz, CA), goat anti mouse-FITC and donkey anti rabbit-FITC (both Jackson lab).

TLR agonists used were Pam_3_CSK_4_ (TLR1/2), poly(I:C) (TLR3), LPS (TLR2 and 4), ultrapure LPS (TLR4), flagellin (TLR5) and CL075 (TLR8; all *In vivo*gen, San Diego, CA).

HDAC inhibitors used were trichostatic acid, valproic acid, romidepsin and pracinostat (all Selleck Chemicals, Houston, TX). JMJD3 inhibitor used was GSKJ4.HCl (Selleck Chemicals) and arginase-1 inhibitor used was CB1158 dihydrochloride (MedChemExpress, Monmouth Junction, NJ).

### Flow Cytometry

Microglia were harvested by incubation with 4 mg lidocaine/ml (Sigma) at 37°C, washed with PBS and kept on ice for all further incubations. Cells were washed in FACS buffer (PBS + 2% BSA), incubated for 10 minutes in FACS buffer containing 10% normal human serum (Sanquin, Leiden, the Netherlands) to prevent a-specific binding of the antibodies, washed with FACS buffer and incubated for 30 minutes with either directly labeled antibodies or unlabeled primary antibodies. If necessary, cells were washed again with FACS buffer and incubated for 30 minutes with secondary antibodies. For intracellular stainings, cells were washed with FACS buffer, permeabilized using the Fix-Perm kit (BD) according to manufacturer’s protocol, and stained as above. Cells were washed with FACS buffer before fixation with 2% paraformaldehyde (USB/Affymetrix, Ohio) in ice-cold PBS for 30 minutes and analyzed on the LSRII (BD) using FACS Diva software (BD).

### Mixed Lymphocyte Reactions

MCSF, GMCSF and GMCSF+IL-4-exposed microglia, and monocyte-derived macrophages and DCs from the same donors were used as stimulator cells in mixed lymphocyte reaction assays against PBMC of genotypically determined Mamu-DR non-matching donor monkeys. Stimulator cells were seeded in 24-well plates for 7 days as described above. On day 7, PBMC from non-matching rhesus monkeys were isolated using LSM (MP Biomedicals) and T cells were further purified using anti-CD3 monoclonal antibody-coated Microbeads on MACS single-use separation columns (Miltenyi Biotech) as described by the manufacturer. Purified CD3^+^ cells were CFSE labeled (BD) according to manufacturer’s protocol. After CFSE labeling, CD3^+^ cells were re-suspended in microglia medium and added to stimulator cells in a stimulator cell:CD3^+^ ratio of 1:10. As positive controls CD3^+^ cells were resuspended in microglia medium supplemented with 40 units recombinant human IL-2/mL (Peprotech) or 5 μg ConA/ml. After 3 days, cells were harvested and analyzed by FACS.

### Cytokine Analysis

IL-12p40 and TNFα levels were measured using commercially available enzyme-linked immunosorbent assay (ELISA) kits according to manufacturer’s protocol (U-Cytech, Utrecht, The Netherlands). Multiplex assays were performed using a customized non-human primate Milliplex Kit (Millipore, Billerica, MA). All cytokines, chemokines and growth factors were analyzed according to manufacturer’s protocol on a Luminex 200 system (Biorad).

### Immunofluorescence

Cells grown on glass coverslips were fixed for 20 min at 4°C with 2% paraformaldehyde, washed with PBS and permeabilized for 15 min in PBS containing 0.1% Triton X-100. After two washes with PBS, slips were incubated for 1h at room temperature with the antibody of interest diluted in PBS, washed twice with PBS, followed by 1h incubation at room temperature with FITC or TRITC-labeled secondary antibodies diluted in PBS (Jackson lab). After extensive washes with PBS, coverslips were mounted using Vectashield/DAPI to visualize the cell nucleus (Vector Laboratories, Burlingame, CA). Images were captured using a Leica fluorescence microscope.

### Real-Time Quantitative RT-PCR

Total cellular RNA was isolated using TriReagent (Sigma) according to manufacturer’s protocol. Subsequently, mRNA was reversely transcribed into cDNA using the Omniscript Reverse Transcription System according to manufacturer’s protocol (Qiagen Benelux, Venlo, The Netherlands) using 0.5 µg RNA as template and 0.25 µg oligo(dT)_15_ primers (Promega Benelux, Leiden, The Netherlands). mRNA levels of target genes were determined by real-time quantitative rt-PCR. Reactions were performed using the CFX96-PCR system (Biorad). Sequences of primer and probe (Probelibrary, Roche, Woerden, the Netherlands) combinations are listed in **Supplementary Table 2**. mRNA expression levels were standardized to GAPDH or β-actin mRNA expression levels using the Pfaffl method (27).

### TransAM ELISA

DNA binding activity of NF-κB was evaluated using the nuclear extract kit and TransAM ELISA kit (both Active Motif, Carlsbad, CA) according to the manufacturer’s instructions. To prepare nuclear extracts, cells were washed with ice-cold PBS. Cells were lysed in cytosolic lysis buffer provided with the kit. After centrifugation at 14,000 g for 30s at 4°C, pellets were resuspended in complete lysis buffer as provided with the kit. After 30 min incubation on ice, nuclei were clarified by centrifugation at 14,000 g for 10 min at 4°C. Supernatants containing nuclear proteins were stored at -80°C and used for further analysis. The NF-κB DNA binding activity was analyzed by TransAM ELISA according to the manufacturer’s protocol. NF-κBp65 protein levels were quantitated using a standard curve of recombinant NF-κBp65 protein (Active Motif).

### Statistics

GraphPad Prism 9 (GraphPad software, San Diego, CA) was used for statistical analyses, *p* values < 0.05 were considered statistically significant.

## Results

### Exposure of Primary Microglia to IL-4 Alters Their Expression Profile of Cell Surface Molecules as Well as Their Potential to Induce T Cell Proliferation

**Figure 1:**
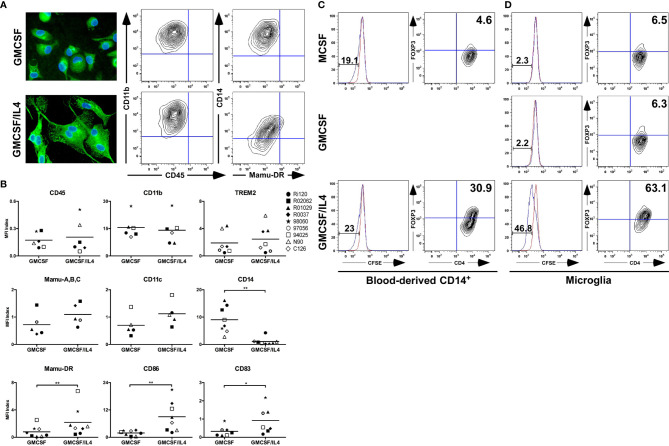
Morphological, phenotypical and functional characterization of primary microglia exposed to IL-4. **(A)** Morphology of primary microglia exposed to GMCSF or GMCSF/IL-4 for 7 days (left panels), as visualized by TREM-2 expression (green). Cell nuclei (blue) were visualized using 4’, 6-diamidino-2-phenylindole (DAPI). Original magnifications x20. Flow cytometric analysis of cell surface expression levels of CD11b, CD45, CD14 and Mamu-DR (right panels). A representative example of at least three different donors is shown. **(B)** Analysis of expression levels of cell surface markers on microglia exposed to GMCSF or GMCSF/IL-4. Levels are expressed as MFI index [(MFI_protein of interest_ – MFI_isotype control_)/MFI_isotype control_]. Different symbols represent different donors and horizontal lines indicate means. *p < 0.05 **p < 0.01 paired t-test. MFI, Mean fluorescent intensity. Capacity of different antigen presenting cell populations (APC) to induce T cell proliferation in mixed lymphocyte reaction assays. Blood-derived CD14+ cells **(C)** or primary microglia **(D)** of the same donors were used as APC. CFSE dilution was measured in CD4+ T cells (left panels, red lines represent CFSE stainings in CD4+ T cells without APC) and Foxp3 expression levels were analyzed (right panels, numbers are % Foxp3+ CD4+ T cells). A representative example of three different donors is shown.

To assess the effects of IL-4 exposure on microglia, primary microglia from adult rhesus monkeys were exposed to GMCSF and IL-4 and compared to microglia that were exposed to GMCSF only for seven days. IL-4-exposed microglia were characterized by a bipolar morphology (**Figure 1A**) and the cultures contained many multinucleated giant cells which were virtually absent in non IL-4-exposed microglia (not shown).

Flow cytometric analysis revealed that exposure to IL-4 did not affect cell surface expression levels of the lineage markers CD11b, CD45 and TREM2, but did significantly reduce the expression levels of CD14 (**Figures 1A, B**). By contrast, IL-4-exposed microglia expressed significantly higher levels of Mamu-DR (the rhesus macaque equivalent of MHC class II), and of the activation markers CD86 and CD83 (**Figure 1B**).

As the cell surface expression profile of IL-4-exposed microglia differed most notably for molecules implicated in the process of antigen presentation, we assessed the functional consequences of this altered expression profile by measuring the potential of IL-4-exposed microglia to stimulate T cell proliferation of Mamu-DR non-matching donor monkeys in mixed lymphocyte reactions (MLR). We compared this ability to non-exposed microglia and classical CD14^+^-derived populations of macrophages and immature dendritic cells (iDC), generated by exposure to MCSF and GMCSF/IL-4 respectively, of the same donors. We also included a population of MCSF-exposed microglia for consistency. CD14^+^-derived macrophages and iDC induced comparable levels of T cell proliferation (19 and 23% respectively) as assessed by CFSE dilution (**Figure 1C**). To our surprise, IL-4-exposed microglia of the same donors were potent inducers of T cell proliferation (47%) both in comparison to MCSF and GMCSF-exposed microglia as well as to CD14^+^-derived macrophages and iDC (**Figure 1D**). Flow cytometric analysis further revealed that IL-4-exposed microglia induced the expression of the regulatory marker Foxp3 in T cells (in 63%, **Figure 1D**), and did so even more potently than iDC (in 31%, **Figure 1C**), suggesting that these T cells might have regulatory potential.

### Toll-Like Receptor-Induced Responses Are Broadly Impaired in IL-4-Exposed Microglia

**Figure 2:**
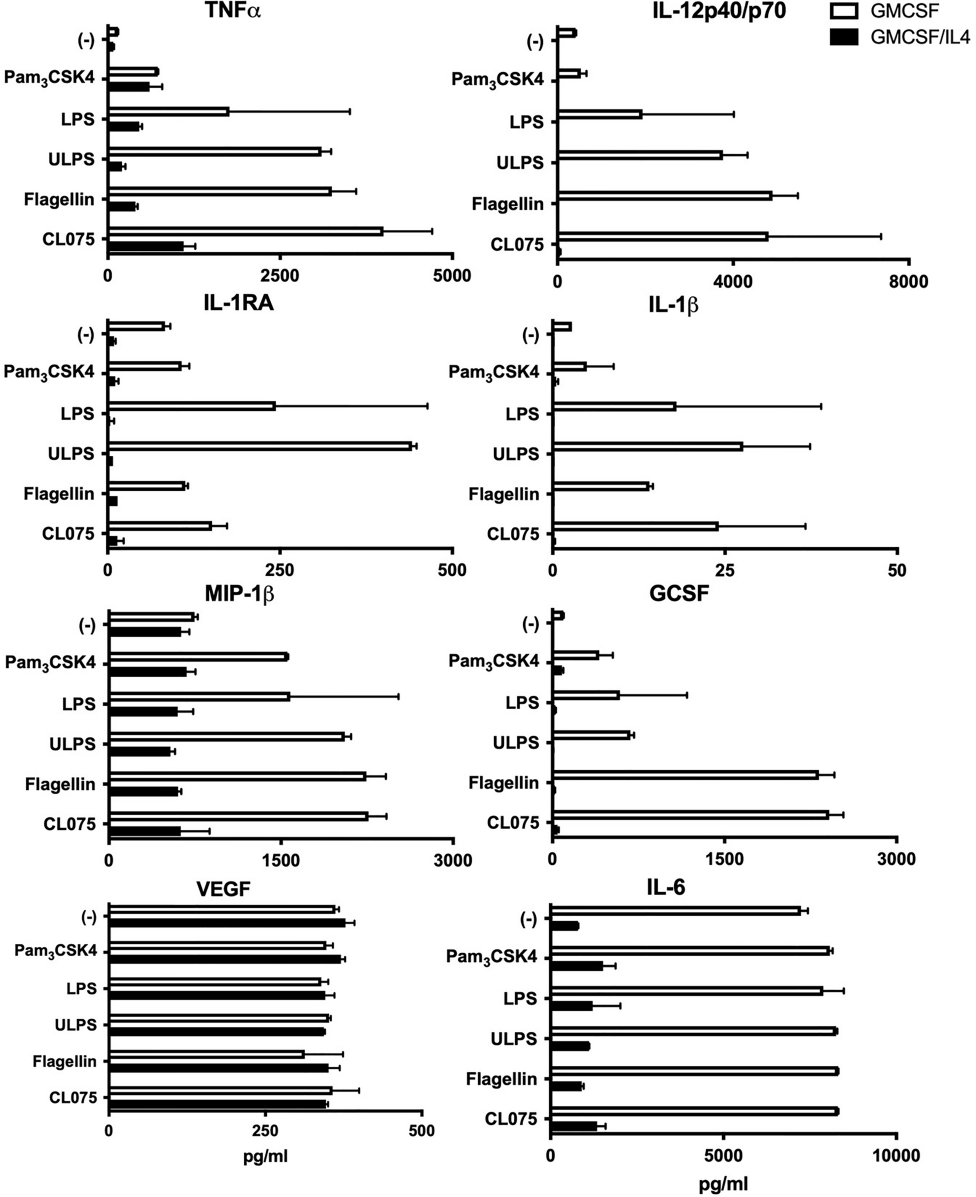
Cytokine production as induced by different TLRs is broadly impaired in microglia exposed to IL-4. GMCSF or GMCSF/IL-4-exposed microglia were stimulated with different TLR ligands for 16h. Cytokine, chemokine and growth factor production in pg/ml. TLR ligands used were 100 ng PAM3CSK4 (TLR1/2), 100 ng LPS/ml (TLR2/4), 100 ng ultrapure LPS/ml (TLR4), 20 µg flagellin/ml (TLR5) or 1 µg CL075/ml (TLR8). A representative example of three different donors is shown, error bars indicate SD.

To assess the effects of IL-4 exposure on microglial innate immune responses, we broadly screened TLR-induced cytokine, chemokine and growth factor responses. We characterized TLR1/2, 2/4, 4, 5 and 8-mediated responses by exposing the cells to PAM_3_CSK4, standard LPS, Ultrapure LPS, flagellin or CL075 respectively. In comparison to non-exposed microglia, TLR-induced levels of IL-1β, IL-1-receptor antagonist, TNFα, IL-12p40/p70, GCSF and MIP-1β were strongly impaired in IL-4-exposed microglia (**Figure 2**). This was irrespective of the TLR ligand used. Also, constitutive IL-6 expression levels were around 10-fold lower in IL-4-exposed microglia as compared to non-exposed microglia whereas constitutive VEGF expression levels were comparable and were not further induced by any of the TLR ligands tested.

**Figure 3:**
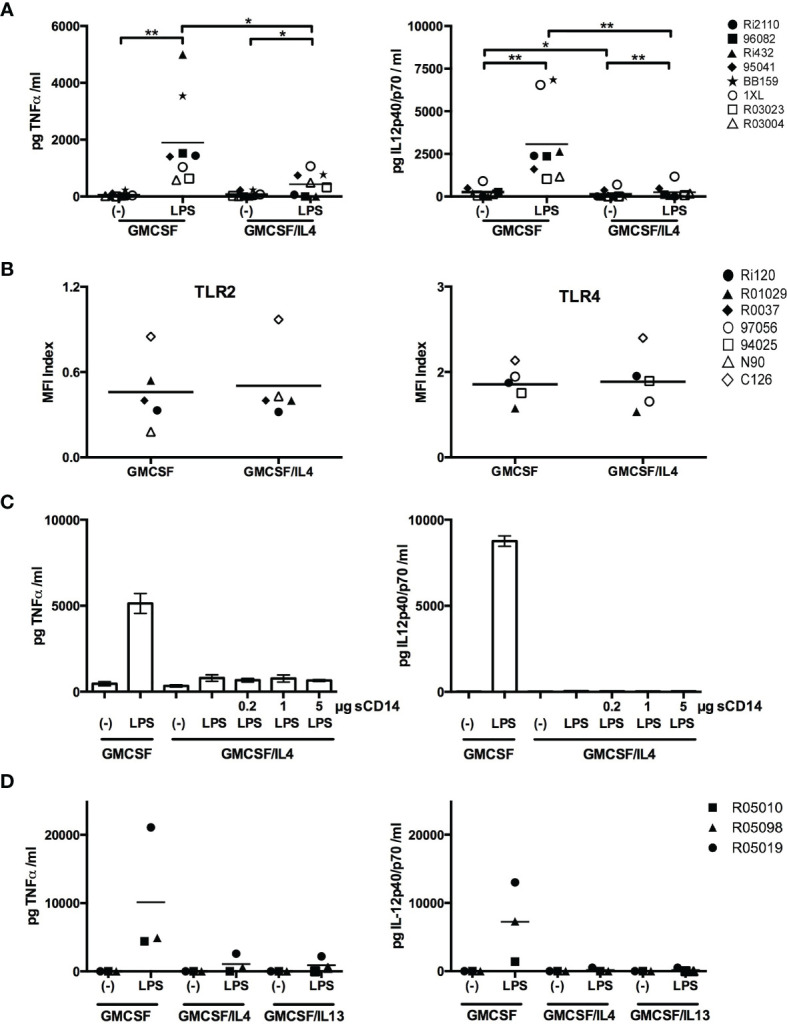
Comprehensive analysis of the impairment of LPS-induced cytokine production in IL-4-exposed microglia. **(A)** GMCSF or GMCSF/IL-4-exposed microglia were stimulated with 100 ng LPS/ml for 16h. TNFα and IL-12p40/p70 levels are expressed in pg/ml, *p < 0.05 **p < 0.01 paired t-test. **(B)** Cell surface expression of TLR2 and TLR4 on GMCSF and GMCSF/IL-4-exposed microglia were assessed by flow cytometry and expressed as MFI index [(MFI_protein of interest_ – MFI_isotype control_)/MFI_isotype control_]. **(C)** GMCSF/IL-4-exposed microglia were stimulated with 100 ng LPS/ml for 16h in the presence of increasing concentrations of soluble CD14. **(D)** TNFα and IL-12p40/p70 production levels (in pg/ml) of microglia exposed to GMCSF, GMCSF/IL-4 or GMCSF/IL-13 that were stimulated with 100 ng LPS/ml for 16h. Different symbols represent different donors and horizontal lines indicate means.

We further characterized the effects of IL-4 exposure by focusing on TLR2/4-induced TNFα and IL-12p40/p70 responses. We confirmed that LPS-induced TNFα and IL-12p40/p70 levels were indeed significantly lower in microglia exposed to IL-4 (**Figure 3A**). These differences could not be attributed to lower cell surface expression levels of TLR on IL-4-exposed microglia, as TLR2 and 4 expression levels were comparable to non-exposed microglia (**Figure 3B**). Alternatively, the decreased expression levels of CD14 on IL-4-exposed microglia (**Figure 1B**) might have negatively affected TLR-induced responses as CD14 is a shared co-receptor of TLR2, 4, and 5 (28). However, the addition of recombinant soluble CD14 did not restore LPS-induced TNFα and IL-12p40/p70 production (**Figure 3C**). Together these data suggest that the impaired TLR responses are not caused by differences in receptor densities, but may rather be caused by IL-4-induced inhibitory effects on TLR-triggered intracellular signaling cascades.

### IL-4 Type 2 Receptors Are Responsible for the Impaired TLR-Induced Cytokine Responses

There are two receptor complexes involved in IL-4-induced signaling. Type 1 receptors consist of an IL-4Rα chain and the common γ chain, and type 2 receptors consist of an IL-4Rα chain and an IL13Rα1 chain (29). IL-4 signals *via* both type 1 and type 2 receptors, whereas IL13 signals only *via* type 2 receptors. To investigate the relative contribution of the receptor subtypes to the IL-4-mediated effects on the impaired TLR-induced responses, we exposed microglia to IL-13 and measured TLR-induced cytokine production. LPS-induced TNFα and IL-12p40/p70 levels were as strongly impaired in microglia exposed to IL-13 as to IL-4 (**Figure 3D**), demonstrating that signaling mediated by IL-4 type 2 receptors is crucial.

### LPS-Induced Cytokine mRNA Levels Are Reduced in IL-4-Exposed Microglia, but NF-κB Translocalization Is Intact

**Figure 4:**
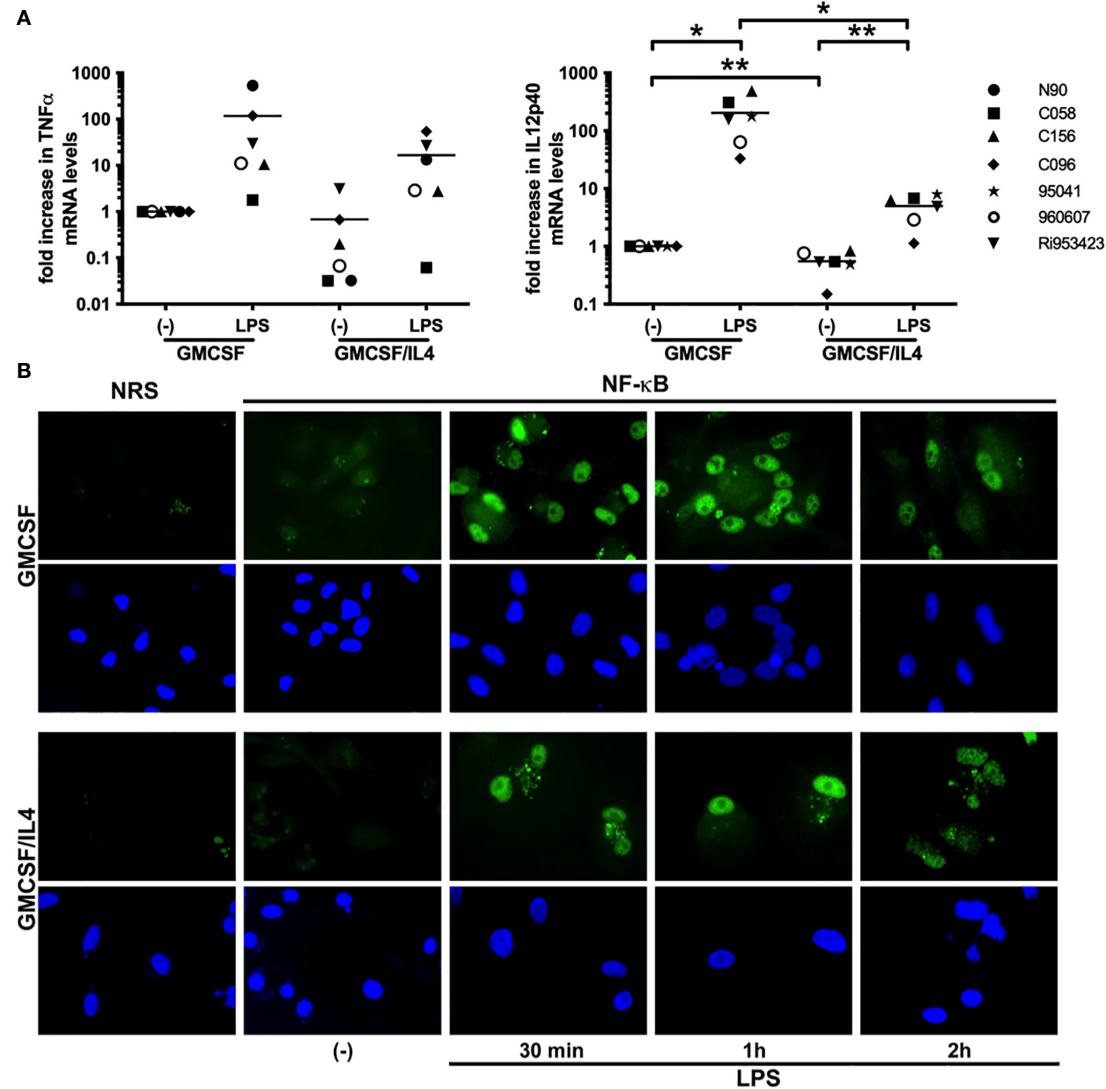
LPS-induced cytokines are impaired at the transcriptional level, but NF-κB translocalization is intact. **(A)** TNFα and IL-12p40-encoding mRNA expression levels in GMCSF and GMCSF/IL-4-exposed microglia stimulated with 500 ng LPS/ml for 16h were quantified by real time RT-PCR. mRNA levels are expressed relative to GAPDH mRNA expression levels, *p < 0.05 **p < 0.01 paired t-test. Different symbols represent different donors and horizontal lines indicate means. **(B)** NF-κB p65 (green) localization in microglia exposed to GMCSF or GMCSF/IL-4 stimulated with 500 ng LPS/ml for 30 min, 1h and 2h [**(B)**, upper panels]. NRS, normal rabbit serum. Cell nuclei (blue) were visualized using 4’, 6-diamidino-2-phenylindole (DAPI, lower panels). Original magnifications x20.

To investigate if IL-4 exposure affected TLR-induced cytokine production at the transcriptional level, we measured LPS-induced TNFα and IL-12p40 encoding mRNA levels by real time PCR. Basal TNFα and IL-12p40-encoding mRNA levels were already reduced to 68% and 55% respectively in IL-4-exposed microglia as compared to non-exposed microglia. Although LPS exposure induced markedly less TNFα encoding mRNA in IL-4-exposed (a mean 16-fold increase) than in non-exposed (a mean 116-fold increase) microglia, this difference was not significant. LPS-induced IL-12p40 encoding mRNA levels on the other hand did differ significantly between IL-4-exposed (a mean 4-fold increase) and non-exposed (a mean 203-fold increase) microglia (**Figure 4A**). Interestingly, also basal IL-12p40 expression levels were significantly lower in IL-4-exposed microglia (about 55% of non-exposed microglia).

As activation and nuclear translocalization of the transcription factor NF-κB are shared features of all TLR-induced responses, we analyzed LPS-induced nuclear translocalization of NF-κB by immunofluorescence. LPS-induced translocalization of NF-κB was intact and comparable both in intensity as well as in kinetics in IL-4-exposed and non-exposed microglia (**Figure 4B**). Together these results demonstrate that although TLR-induced cytokine responses are affected at the transcriptional level in IL-4 exposed microglia, the shared upstream NF-κB signaling cascade appears intact.

### Nuclear Extracts From IL-4-Exposed Microglia Contain Significantly Less Activated NF-κB

**Figure 5:**
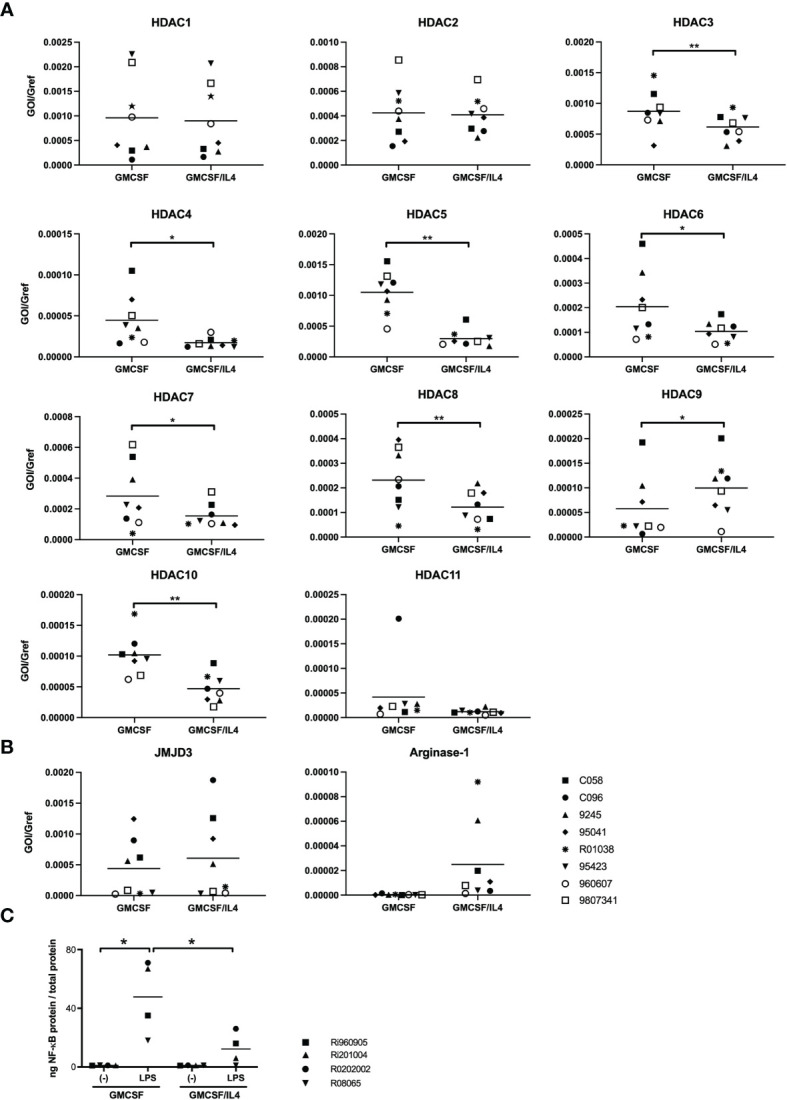
Expression profiles of epigenetic modulators and analysis of LPS-induced binding of NF-κB to target DNA. **(A)** HDAC-encoding mRNA expression levels in GMCSF and GMCSF/IL-4-exposed microglia were quantified by real time RT-PCR. mRNA levels are expressed relative to GAPDH mRNA expression levels, *p < 0.05 **p < 0.01 paired t-test. **(B)** JMJD3 and arginase-1-encoding mRNA expression levels in GMCSF and GMCSF/IL-4-exposed microglia were quantified by real time RT-PCR. mRNA levels are expressed relative to GAPDH mRNA expression levels. **(C)** The capacity of nuclear NF-κB to bind to its DNA consensus sequence was measured in nuclear lysates of microglia exposed to GMCSF or GMCSF/IL-4 stimulated with 500 ng LPS/ml for 1h. *p < 0.05 paired t-test. Different symbols represent different donors and horizontal lines indicate means.

We therefore turned our focus on possible differences in histone acetylation. This plays an important role in the regulation of gene expression as hyperacetylated chromatin is transcriptionally active, whereas hypoacetylated chromatin is silent. Different expression patterns of epigenetic regulators might therefore underlie the broad impairment of TLR-induced cytokines in IL-4-exposed microglia, as has been described for macrophages (30–32). We first profiled the expression patterns of all known histone deacetylates (HDACs) in non-exposed and IL-4-exposed microglia (**Figure 5A**). Higher HDAC expression levels in IL-4-exposed microglia would be consistent with possible epigenetic repression of target genes. Whereas expression levels of HDAC1, 2 and 11-encoding mRNAs did not differ significantly between non-exposed and IL-4-exposed microglia, expression levels for HDAC 3-8 and HDAC10 were to our surprise all significantly higher in non-exposed microglia. Only HDAC9 was expressed at significantly higher levels in IL-4-exposed microglia. These results render it unlikely that differential expression of HDACs underlie the impaired TLR-induced cytokine responses in IL-4-exposed microglia. This was in line with results using HDAC inhibitors (trichostatic acid, valproic acid, romidepsin and pracinostat) in attempts to rescue LPS-induced TNFα and IL-12p40/p70 responses in IL-4-exposed microglia, which had no measurable effects (data not shown).

We next investigated the possible involvement of another previously described epigenetic modulator in macrophage differentiation. JMJD3 is a H3K27 demethylase, that has been demonstrated to be pivotal for microglial ‘M2’ polarization (33). Although expression levels of JMJD3-encoding mRNA were comparable between non-exposed and IL-4-exposed microglia (**Figure 5B**), mRNA expression levels of arginase-1, that are regulated by JMJD3, appeared indeed to be, non-significantly, enhanced in IL-4-exposed microglia (**Figure 5B**). Enhanced expression levels of arginase-1 are thought to compete with iNOS for the available L-arginase (their shared substrate) thereby affecting intracellular levels of available NO. However, specific inhibition of JMJD3 or arginase-1 activity by exposure of microglia to GSKJ4.HCl or CB1158 dihydrochloride respectively did not rescue LPS-induced TNFα or IL12p40/p70 responses in IL-4-exposed microglia (data not shown).

As we did not find any indications for epigenetic repression of LPS-induced transcription of TNFα or IL12p40 in IL-4-exposed microglia, we further analyzed the functionality of the nuclear NF-κB. We therefore assessed the levels of NF-κB p50/65 heterodimers capable of binding to its DNA consensus site before and after LPS stimulation, in nuclear extracts that were standardized for total protein content. Although LPS-induced signaling led to a marked increase of levels of activated NF-κB in the nuclei of either cell population, nuclei from IL-4-exposed microglia contained significantly lower levels of NF-κB capable of DNA binding than non-exposed microglia, by about a 4-fold (**Figure 5C**).

## Discussion

In this study, we demonstrate that exposure of primary adult rhesus monkey microglia to IL-4 affects their cell surface protein expression profile as well as their potential to induce the proliferation of T cells with a regulatory signature, which is largely in line with earlier studies (8, 24, 34–36). In particular, we here delineate the effects of IL-4 exposure on TLR-induced responses. Whereas it has already been reported that IL-4 exposure of primary human microglia skews TLR4-induced cytokine production from a predominantly pro-inflammatory towards an anti-inflammatory profile, we here demonstrate that TLR-mediated responses are broadly and also quantitatively affected.

Mechanistically, we show that IL-4 type 2 receptor-mediated signaling is sufficient to cause inhibition of TLR-mediated responses, although we have not formally ruled out a role for IL-4 type 1 receptor-mediated signaling. IL-4 type 2 receptor-mediated signaling induces the activation of different signal transduction pathways, such as phosphatidylinositol-4,5-bisphosphate 3-kinase (PI3K) as well as the activation of the transcription factors signal transducer and activator of transcription (STAT)-3 and STAT-6, that can all modulate NF-κB activation. IL-4-induced phosphorylation of STAT-3 and STAT-6 has been described to repress transcription of NF-κB-activated genes mainly *via* inhibition of NF-κB nuclear translocalization (37–39). Although NF-κB translocalization was not quantitatively assessed in this study, our results strongly suggest that NF-κB translocalization was intact in IL-4-exposed microglia while LPS-induced cytokines responses were impaired at the transcriptional level.

In other macrophages, epigenetic repression of target genes has been described after IL-4 exposure. However, our analyses of expression patterns of epigenetic regulators, as well as rescue experiments using a variety of inhibitors, did not yield any evidence for epigenetic modulation to underlie the impaired LPS responses in IL-4-exposed microglia. We therefore further analyzed the functionality of the nuclear NF-κB in the different cell populations by assessing the levels of NF-κB p50/65 heterodimers capable of binding to its DNA consensus site before and after LPS stimulation, in nuclear extracts that were standardized for total protein content.

Our results show that nuclear extracts from IL-4-exposed microglia contained significantly lower levels of NF-κB p50/p65 heterodimers capable of binding to its DNA consensus site than non-exposed microglia. This could of course be due to overall lower expression levels of NF-κB p50 or p65 in IL-4-exposed microglia, for which we did not find any evidence. Alternatively, STAT-6 has been described to heterodimerize with NF-κB p50 subunits (40). Such heterodimers do not trigger transcription of NF-κB-responsive genes, while they do compete with NF-κB p50/p65 heterodimers for available NF-κB DNA binding sites which would be consistent with our results. In addition, DNA binding of NF-κB p50/p65 is also regulated by phosphorylation and acetylation of the p65 subunit (41). The balance between acetylation and de-acetylation regulates the overall duration of DNA binding, where de-acetylation of NF-κB p65 leads to reassembly of NF-κB with IκB, followed by nuclear export (42–44). Interestingly, PI3K-signaling has been shown to affect NF-κB acetylation *via* GSK3β inactivation in microglia (45), and STAT-6 may limit the acetylation of NF-κB *via* the induction of Kruppel-like factor 4 (KLF4) (46). More recent studies have unveiled that, in macrophages the repressor function of STAT6 on a subset of IL-4-repressed genes is HDAC3 dependent (47, 48). Future studies in our lab are aimed at further elucidation of the pathways involved.

Our results may be of particular interest for gliomas, where the use of TLR ligands to trigger glioma-infiltrating microglia (GIM) to provoke anti-tumor responses is currently under evaluation in clinical trials (49–51). GIM are characterized by impaired TLR-induced cytokine responses despite normal TLR expression levels (52–54). Different studies have reported on the importance of IL-4 and GMCSF in glioma development and/or progression (55–57), showing an enhanced risk and worse outcome of GBM for people that carry certain polymorphisms in IL-4R and STAT-6 gene loci (12–15). However, the underlying mechanisms still remain largely unknown. Our results would suggest that therapeutic triggering of innate immune responses by GIM might benefit from simultaneous inhibition of IL-4 type 2 receptor-mediated signaling.

## Data Availability Statement

The raw data supporting the conclusions of this article will be made available by the authors, without undue reservation.

## Author Contributions

EZ-S, CP, RT, JV, LS, and EP conducted the experiments, PV and SA made materials available and contributed to the discussions. EZ-S, CP, RT, and JB conceived the experiments and wrote the paper. All authors contributed to the article and approved the submitted version.

## Conflict of Interest

The authors declare that the research was conducted in the absence of any commercial or financial relationships that could be construed as a potential conflict of interest.

## Publisher’s Note

All claims expressed in this article are solely those of the authors and do not necessarily represent those of their affiliated organizations, or those of the publisher, the editors and the reviewers. Any product that may be evaluated in this article, or claim that may be made by its manufacturer, is not guaranteed or endorsed by the publisher.
