# Supplementary material for: Exposure of Microglia to Interleukin-4 Represses NF-κB-Dependent Transcription of Toll-Like Receptor-Induced Cytokines
Source: Front Immunol. 2021 Nov 22;12:771453. doi: 10.3389/fimmu.2021.771453 (PMC8645606; doi:10.3389/fimmu.2021.771453)
Supplement: Supplementary file 1 [file Table_1.doc]

| **Animal identification no.** | **Age (yr)** | **Sex** | **Weight (kg)** | **Origin** |
| --- | --- | --- | --- | --- |
| 1XL | 25 | F | 9.6 | India |
| 8765 | 22 | F | 9.5 | Mix |
| 9245 | 18 | F | 5.6 | Mix |
| 94025 | 15 | F | 7.9 | India |
| 95041 | 14 | F | 8.9 | India |
| 96082 | 13 | F | 7.2 | India |
| 97056 | 6 | M | 5.4 | India |
| 98060 | 9 | M | 11.9 | Mix |
| BB159 | 9 | F | 6.3 | Unknown |
| C028 | 25 | F | 9.0 | China |
| C033 | 22 | M | 9.3 | China |
| C058 | 15 | F | 7.1 | China |
| C096 | 15 | F | 7.5 | China |
| C126 | 15 | F | 6.5 | China |
| C156 | 15 | F | 7.7 | China |
| N90 | 15 | F | 8.2 | China |
| R0037 | 9 | M | 11.6 | India |
| R01029 | 7 | F | 6.0 | India |
| R0138 | 5 | M | 8.8 | India |
| R02022 | 12 | F | 8.3 | India |
| R02062 | 5 | M | 8.8 | India |
| R03004 | 7 | M | 7.2 | India |
| R03016 | 7 | F | 5.2 | India |
| R03023 | 7 | M | 10.4 | India |
| R05010 | 6 | M | 11.9 | Mix |
| R05019 | 7 | M | 9.4 | Mix |
| R05098 | 7 | M | 13.3 | Mix |
| R08065 | 3 | M | 3.3 | India |
| Ri120 | 14 | M | 9.2 | India |
| Ri201004 | 9 | F | 6.5 | China |
| Ri2110 | 9 | F | 5.8 | Unknown |
| Ri432 | 12 | F | 6.7 | India |
| Ri953423 | 15 | M | 13.7 | China |
| Ri960607 | 14 | M | 12 | China |
| Ri960905 | 11 | M | 14.9 | China |
| Ri9807341 | 12 | M | 13.1 | China |
| Ri202062 | 9 | F | 5.8 | China |

**Supplementary Table 1**. Animal specifications.
