# Supplementary material for: Exposure of Microglia to Interleukin-4 Represses NF-κB-Dependent Transcription of Toll-Like Receptor-Induced Cytokines
Source: Front Immunol. 2021 Nov 22;12:771453. doi: 10.3389/fimmu.2021.771453 (PMC8645606; doi:10.3389/fimmu.2021.771453)
Supplement: Supplementary file 2 [file Table_2.docx]

| Target | Forward primer (5’-3’) | Reverse primer (5’-3’) | Probe |
| --- | --- | --- | --- |
| TNFα | aagcctgtagcccatgttgt | gctggttatctgtcagctcca | ccaggagg |
| IL-12p40 | ccacattcctacttctccctga | accgtggctgaggtcttgt | tccaggtc |
| HDAC1 | agatgaccaagtaccatagtgacg | agtcctcaccaacgttgaatc | ctccatcc |
| HDAC2 | cagatcgtgtaatgacggtatca | ccttttccagcaccaatatcc | ttcctggc |
| HDAC3 | gacctatgacaggactgatgagg | gaactcattgggtgcctctg | ctcctctg |
| HDAC4 | cagcaactgcacatgaacaa | agcagagcctggtgctca | ctcctctg |
| HDAC5 | tctcacctgacgtggtccta | ctggtcaagtggccaaaac | cccagcag |
| HDAC6 | agttcaccttcgaccaggac | ccagaacctaccctgctcat | ctccatcc |
| HDAC7 | tgtcaatgtggcctggact | caatgggcatcacgactgt | gcagccag |
| HDAC8 | tctggaggctggcatcat | aaatttccgtcgcaatcgta | ggacagca |
| HDAC9 | agtgccatcccagctcaat | gaagacgcagggatgctg | cagaggaa |
| HDAC10 | tgaccctgagctggtgct | aggtgggcgaagcactct | cagcagga |
| HDAC11 | acacgaggcgctatcttaatg | agcaccttcctctgcacaa | cttcctcc |
| JMJD3 | gcgaccctgatttgttcaag | ctgtacctggcagtgcttca | tctgctgc |
| Arginase-1 | cctcctgaaggaactaaaaggaa | cagatatgcagggcgtca | ttctcctg |
| GAPDH | caacgaccactttgtcaagc | gtggtcccgggggtcttac | ccaccacc |
| β-actin | gcccagcacgatgaagat | cgccgatccacacagagta | aggaggag |

**Supplementary Table 2.** Primer and probe sequences.
